# Fluoride passivation of ZnO electron transport layers for efficient PbSe colloidal quantum dot photovoltaics

**DOI:** 10.1007/s12200-023-00082-3

**Published:** 2023-10-27

**Authors:** Jungang He, You Ge, Ya Wang, Mohan Yuan, Hang Xia, Xingchen Zhang, Xiao Chen, Xia Wang, Xianchang Zhou, Kanghua Li, Chao Chen, Jiang Tang

**Affiliations:** 1 Hubei Key Laboratory of Plasma Chemistry and Advanced Materials, Hubei Engineering Technology Research Center of Optoelectronic and New Energy Materials, School of Materials Science and Engineering, Wuhan Institute of Technology, Wuhan, 430205 China; 2 Wuhan National Laboratory for Optoelectronics (WNLO), School of Optical and Electronic Information, School of Integrated Circuits, Huazhong University of Science and Technology, Wuhan, 430074 China

**Keywords:** Zinc oxide, Surface passivation, Band alignment, Quantum-dot solar cells

## Abstract

Lead selenide (PbSe) colloidal quantum dots (CQDs) are suitable for the development of the next-generation of photovoltaics (PVs) because of efficient multiple-exciton generation and strong charge coupling ability. To date, the reported high-efficient PbSe CQD PVs use spin-coated zinc oxide (ZnO) as the electron transport layer (ETL). However, it is found that the surface defects of ZnO present a difficulty in completion of passivation, and this impedes the continuous progress of devices. To address this disadvantage, fluoride (F) anions are employed for the surface passivation of ZnO through a chemical bath deposition method (CBD). The F-passivated ZnO ETL possesses decreased densities of oxygen vacancy and a favorable band alignment. Benefiting from these improvements, PbSe CQD PVs report an efficiency of 10.04%, comparatively 9.4% higher than that of devices using sol-gel (SG) ZnO as ETL. We are optimistic that this interface passivation strategy has great potential in the development of solution-processed CQD optoelectronic devices.

**Graphical Abstract:**

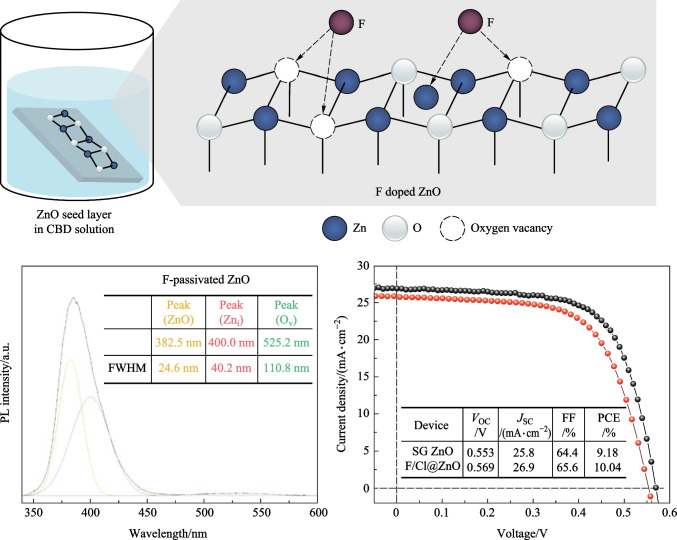

**Supplementary Information:**

The online version contains supplementary material available at 10.1007/s12200-023-00082-3.

## Introduction

Solution-processed colloidal quantum dots (CQDs) are promising candidates for optoelectronic applications, including photodetectors [1, 2], photovoltaics [3, 4] and light-emitting diodes [5, 6]. Among these CQDs, PbSe CQDs possess a broad response range (0.3 − 2 eV) [7, 8], large exciton Bohr radius (46 nm) [9, 10], and strong multiple exciton generation (~ 120% MEG) [11, 12], making them extremely suitable for application in infrared photodetectors and tandem solar cells. However, the development of PbSe CQD photovoltaics is sluggish because of poor air stability [13, 14]. Thanks to the rapid advances of metal halide ligands and solution phase ligand exchange process [15, 16], the air stability of PbSe CQDs significantly increases [3, 4]. Once these CQDs are assembled into solar cells with the architecture of ITO/ZnO/lead halides-capped PbSe CQDs/PbS-1,2-ethanedithiol (EDT)/Au, the power conversion efficiency (PCE) of PbSe CQD solar cells can be up to 11.6% [4], which is the highest efficiency for all reported PbSe CQD solar cells.

From the description above, it is clear that progress of PbSe CQDs solar cells has been achieved through CQD surface passivation using metal halides and solution phase exchange process [3, 4, 17]. In view of these developments, further improvement for device performance can focus on the optimization of electron transport layer (ETL) and hole transport layer (HTL). Until now, EDT-treated PbS CQD HTLs have been important building blocks for PbSe CQD solar cells because they have high-matching band alignment and low lattice mismatch with PbSe CQD active layers; this benefits efficient extraction and transport of hole carriers from CQD active layers [18–20]. Ahmad and co-workers demonstrated the effect of EDT-treated PbS CQD HTLs on the performance of PbSe CQD solar cells [3]. It was reported that EDT-treated PbS CQD HTLs with moderated oxidization would provide appropriate hole concentration and matched band alignment, whereas EDT-treated PbSe CQD HTLs did not have these advantages due to the compensation effect from in-situ halide precursor during the synthetic process. Owing to in-situ halide doping, the Fermi level of EDT-treated PbSe CQDs is shifted upwards, resulting in low hole extraction efficiency and device performance. In view of these issues, researchers have focused on lead chalcogenide CQD photovoltaics, still employing EDT-treated PbS CQDs as HTLs. Hence, we should pay more attention to the optimization of ETLs in PbSe CQD solar cells. It is not doubted that the performance of lead chalcogenide CQD solar cells would also improve by the optimization of HTL [21, 22]. However, as the efficient PbSe CQD solar cells are reported using the architecture of ZnO/PbI_2_-capped PbSe CQDs/EDT-PbS CQDs, we currently focus on the optimization of ETL so that the architecture of PbSe CQD solar cells has little change.

Right now, several ETLs are widely used in photovoltaics, such as titanium oxide (TiO_2_), tin oxide (SnO_2_), and zinc oxide (ZnO). Compared with other ETLs, ZnO ELTs have become one of the most important building blocks in lead chalcogenide CQD photovoltaics because of low-temperature processing, low work function, and high electron mobility [3, 4, 18, 19]. The device performances of PbS CQD and PbSe CQD solar cells using ZnO as ELTs have been increased to 15.4% and 11.6% [4, 22], respectively. Despite rapid progresses that lead chalcogenide CQD photovoltaics have achieved by using ZnO as ETLs, the quality of ZnO should be improved further [23–25]. The main reason is that the electrical and optical properties of ZnO can be affected by oxygen vacancies, which can capture electrons and induce barriers in the ZnO energy band, hindering carrier extraction and degrading the performance of CQD solar cells [26, 27].

In this study, we tried to use F anions to passivate the oxygen vacancies of ZnO. Owing to their small ionic radius (1.33 Å) and strong electronegativity, F^−^ ions can easily enter lattice sites of ZnO and passivate all the defects [28]. Furthermore, previous studies have demonstrated that F-passivated ZnO has low lattice strain, low defect concentration, confined electronic perturbation, and reduced electron scattering [29, 30]. Hence, the mobility and resistivity of F-passivated ZnO was 46.2 cm^2^/(V·s) and 7.95 × 10^−4^ Ω·cm, respectively, whereas the mobility and resistivity of Cl-passivated ZnO was 27.35 cm^2^/(V·s) and 6.344 × 10^−4^ Ω·cm, respectively [30]. Our results demonstrated that the oxygen vacancies are almost completely passivated. In view of this finding, we inferred that the growth and defect passivation of ZnO was simultaneous during CBD deposition, whereas the growth and defect passivation of ZnO reported by Choi et al. [27] involved two steps. They first synthesized ZnO nanoparticles (NPs), and then used NaCl/methanol for surface passivation of ZnO NPs. Owing to large surface-to-volume ratio and steric hindrance, the defect density of ZnO prepared by Choi et al. was higher than that of ZnO prepared by using our strategy. Subsequently, the F-passivated ZnO (F@ZnO) was configured into PbSe CQD solar cells as ETLs, the device efficiency approached to 10.04%, comparatively 9.4% higher than that of devices using sol-gel (SG) ZnO as ETLs (9.18%).

## Results and discussion

**Fig. 1:**
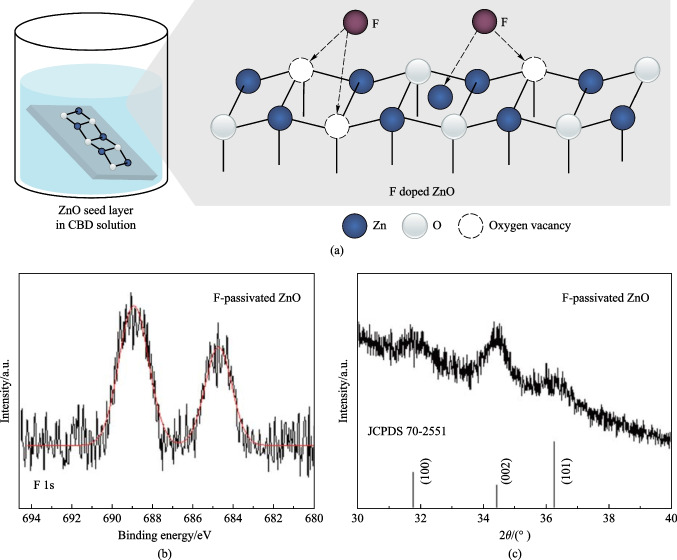
**a** Schematic illustration of the F passivation mechanism of ZnO through chemical bath deposition. **b** F 1s XPS spectrum of ZnO. **c** XRD pattern of F passivated ZnO

As ZnO is difficult to grow heterogeneously on ITO substrates [28], we initially deposited a ZnO seed layer onto ITO. This seed layer was prepared by spin-coating ZnO sol-gel (SG) solution onto ITO and annealing at high temperature [3]. The ZnO seed layer prepared using this process had a degree of crystallinity, which is beneficial for the oriented growth of F-passivated ZnO in CBD solution, as was demonstrated in the XRD results. Then this seed layer was immersed into a precursor solution containing deionized (DI) water, zinc nitrate (Zn(NO_3_)_2_), ammonia (NH_3_), ammonium citrate (C_6_H_5_O_7_(NH_4_)_3_), and ammonium fluoride (NH_4_F). The mixture solution was heated up to 70 ℃ and kept at this temperature for several minutes until a desired thickness of F@ZnO layer was obtained. The detailed information for the preparation of ZnO layer would be introduced in the “Experimental section” (Sect. 4). Figure 1a shows the schematic diagram of this chemical bath deposition method. In order to investigate the binding affinity of F to ZnO, X-ray photoelectron spectroscopy (XPS) was employed, as shown in Fig. 1b. The result indicated that F successfully incorporated into ZnO, the two F-1s peaks located at 689.0 and 684.7 eV, indicating that the F ions appeared both in the lattice and on the surface of ZnO. This result was consistent with previous results that F ions can appear both in the lattice and on the surface of ZnO because the radius of F ions (1.36 Å) is similar to that of oxygen ions (1.40 Å) [30]. According to the literature, oxygen defects (*V*_O_) were the dominant defects due to the low formation energy [30]. In view of this, *V*_O_ could easily form on the surface and lattice sites. As the ionic radius of F^−^ was close to that of O^2−^, and also due to the strong electronegativity of F^−^, F^−^ anions could easily enter into ZnO and passivate *V*_O_ defects. Moreover, the Zn 2p spectra of F@ZnO show a slight peak shift towards a higher binding energy (from 1021.2 to 1021.5 eV) compared to those of ZnO, as depicted in Fig. S1 in the Supporting Information. This shift suggests that F was bound directly to Zn, given that the reported binding energies of Zn–O and Zn-F are 1021.1 and 1021.4 eV [28, 31], respectively. Subsequently, an X-ray diffractometer (XRD) was utilized to investigate the phase structure of the F@ZnO layer. Figure 1c shows that the F@ZnO layer had the main peaks (100), (002) and (101) at around 31.8°, 34.4°, and 36.3° consistently with the standard PDF card of ZnO (JCPDS: 70-2551). This result indicated that the introduction of F anions did not change the crystal lattice spacing of ZnO, as shown in Fig. S1c. Additionally, Fourier Transform Infrared Spectrometer (FTIR) was employed to investigate the residual chemical components in ZnO. Fig. S1d shows that there was no difference between either SG ZnO and F-passivated ZnO. Then, the atomic force microscope (AFM) was used to check the thickness of the as-prepared layer and the roughness of F@ZnO (8.3 nm) was found to be the same as that of SG ZnO (8.5 nm), indicating that ZnO passivated by F had high quality morphology, as shown in Fig. S2.

**Fig. 2:**
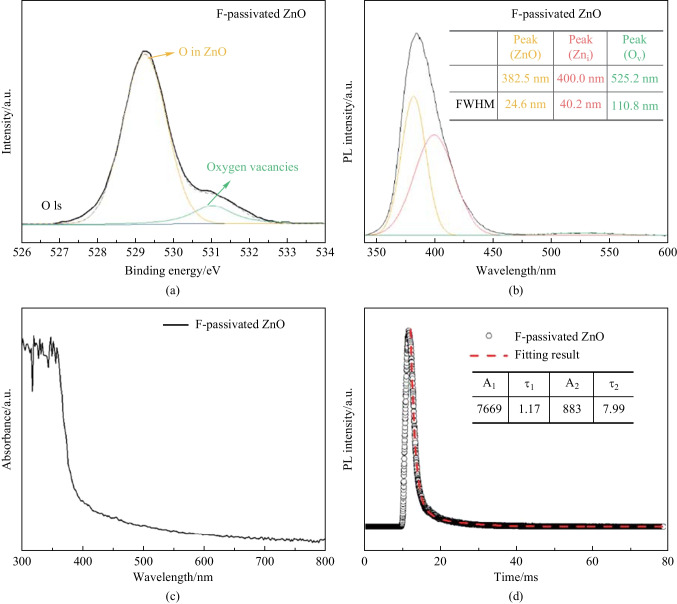
**a** O 1s XPS spectrum of F passivated ZnO. **b** PL spectra of F passivated ZnO. The inserted table is the fitting FWHM results of ZnO defects. **c** Absorbance spectrum of F-passivated ZnO. **d** Transient PL spectra of F-passivated ZnO

Since F@ZnO was successfully obtained, we here discuss F passivation on the density improvement of oxygen vacancies. O-1s spectra in Fig. 2a shows that the intensity of oxygen vacancies was smaller than that of SG ZnO in Fig. S3a, indicating that F^−^ ions with small radius and strong electronegativity had superior ability to passivate the oxygen vacancies of ZnO. It is well known that oxygen vacancies would generate a broad luminescence near 530 nm wavelength in ZnO [27], hence photoluminescence (PL) spectroscopy was carried out to investigate the defect density of F@ZnO. Figure 2b shows that the PL intensity of F@ZnO around 530 nm wavelength largely decreased, as demonstrated in Fig. 2a. In order to analyze the change of defect density, we used the Gaussian function to fit the PL results. The results showed that the full-width half-maximum (FWHM) of the PL peak of interstitial zinc decreased to 40.2 nm, comparatively 48% lower than that of SG ZnO ETLs. Meanwhile, the FWHM of the PL peak of intrinsic zinc increased to 24.6 nm, comparatively 20% higher than that of SG ZnO ETLs. Additionally, transient PL spectra were used for the characterization of carrier life time. Figure 2d shows that F-passivated ZnO had time constants of 1.17 and 7.99 ms. Fig. S5d shows that F/Cl-passivated ZnO had time constants of 1.21 and 8.07 ms, indicating that the carrier life time of F-passivated ZnO showed little change using small amount of Cl ions. These results demonstrated that the time constants of F and F/Cl-passivated ZnO were lower than those of SG ZnO (1.46 and 10.9 ms) in Fig. S3d, indicating that the carrier life time of ZnO could be improved by F passivation. These results showed that F^−^ ions could passivate interstitial zinc and oxygen vacancy of ZnO, which confirmed the result demonstrated in Fig. 1b. All these results showed that F@ZnO possessed unique optical property with few oxygen vacancies.

**Fig. 3:**
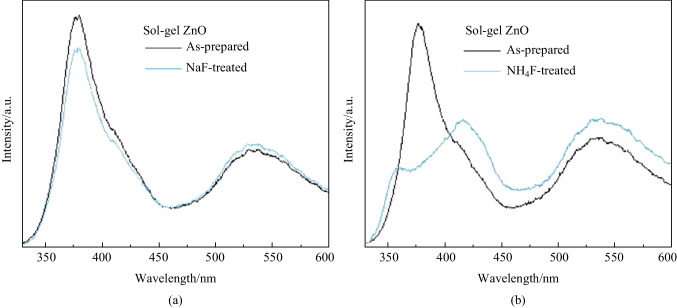
PL spectrum of ZnO treated by different F-based salts. **a** NaF treatment. **b** NH_4_ treatment

In order to ensure the passivation effect of our protocol on oxygen vacancies, we carried out a control experiment. NaF and NH_4_F were dissolved in methanol and used for the surface treatment of SG ZnO. The molar concentration of these salts was same as that of the salts used in our CBD method. Figure 3a shows that there was no change when NaF was used for the surface treatment of SG ZnO. Then, we used NH_4_F for further investigation. Figure 3b demonstrates that the defect density of interstitial zinc and oxygen vacancies of ZnO largely increased. In addition, the PL intensity of ZnO near 358 nm wavelength largely decreased. The main reason was that NaF/methanol was neutral, while NH_4_F/methanol was acidic [32, 33]. Hence, the quality of ZnO would be severely deteriorated when treated by NH_4_F/methanol. Based on the above discussion, we ensured that our protocol was powerful process for the passivation of oxygen vacancies in ZnO. NH_4_F simply used for surface treatment would severely deteriorate the quality of ZnO.

**Fig. 4:**
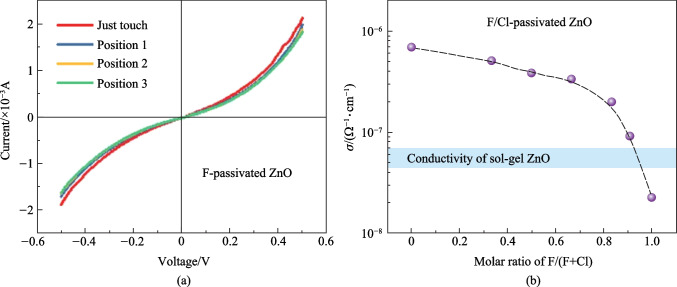
Conductivity study of ZnO treated using different molar ratios of F/Cl under dark. **a** Current − voltage characteristic of F passivated ZnO. **b** Conductivity of ZnO passivated with different molar ratios of F/Cl

After a detailed study to optical properties of F@ZnO, we investigated its electrical property. Aluminum electrodes were evaporated onto F@ZnO for ohmic contact, the structure is shown in Fig. S4a. The schematic diagram for conductivity measurement of ZnO is shown in Fig. S4b. To ensure the reliability of the measured data, the probe was pressed with different forces on Al electrodes so that the probe could have close contact with the electrodes. Figure 4a demonstrates that no matter what the position of the probe was, the current generated in F@ZnO had no obvious change, whereas the current in the case of SG ZnO had obvious change when the probe moved to different positions. These results indicated that F@ZnO formed stable contact with Al electrodes, whereas the contact between SG ZnO and electrodes was weak. Compared with the current for SG ZnO at − 0.5 V, the current for F@ZnO at − 0.5 V was stable at 0.75 mA, as shown in Fig. 3a and Fig. S4d. These results showed that the conductivity of F@ZnO (2.26 × 10^−8^ Ω^−1^⋅cm^−1^) was smaller than that of SG ZnO (6.85 × 10^−8^ Ω^−1^⋅cm^−1^). To improve the conductivity of F@ZnO, we tried to add ammonium chloride (NH_4_Cl) during the process of CBD deposition. This was motivated by the unique electrical property of Cl-passivated ZnO (Cl@ZnO) in Fig. S4c. The molar ratios of F/Cl were set at 1:0, 10:1, 5:1, 2:1, 1:1, 1:2, and 0:1. We found that the conductivity of F/Cl@ZnO (9.16 × 10^−8^ Ω^−1^⋅cm^−1^) was close to that of SG ZnO when the molar ratio of F/Cl was 10:1, as shown in Fig. 3b. The effect of Cl on the density of oxygen vacancies on ZnO, the PL spectrum as shown in Fig. S5 indicates that the intensity of the 530 nm PL peak had little increased. However, as the FWHM of F/Cl@ZnO was almost equal to that of F@ZnO, we inferred that the passivation effect of F on ZnO had no obvious change with a small amount of added Cl.

**Fig. 5:**
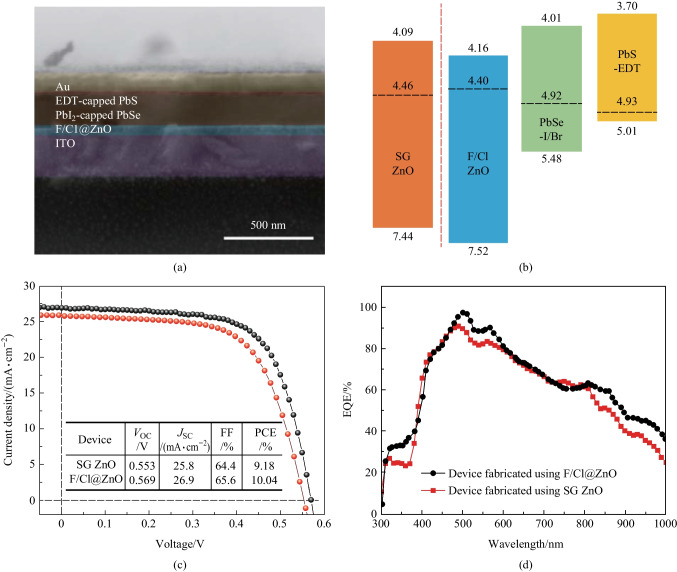
**a** Cross-sectional SEM image of representative PbSe CQD solar cells. **b** Band alignments of the devices prepared using F/Cl@ZnO and sol-gel (SG) ZnO. **c** Current density − voltage (*J − V*) characteristics of devices prepared using F/Cl@ZnO and SG ZnO and performance parameters under AM 1.5G solar illumination. **d** EQE spectra of devices prepared using F/Cl@ZnO and SG ZnO as ETLs, respectively

Finally, F@ZnO was used as ETLs for the construction of PbSe CQD solar cells. The cross section of solar cells is shown in Fig. 5a. According to the SEM image, the thickness of F@ZnO, PbSe CQD active layer, and EDT-PbS CQD HTL were about 40, 200, and 40 nm, respectively. In order to assess the energy band alignment of F@ZnO, transmission spectra and ultraviolet photoelectron spectra were carried out. The bandgap of F@ZnO could be obtained by extracting data from transmission spectrum and then calculated by using Tauc plots [34–37]. The Tauc plots of ZnO layers showed the bandgaps of SG ZnO and F@ZnO (3.35 eV) were similar to those found in a previous study [38]; see Fig. S6b. Additionally, the absorbance band edge of SG ZnO and F@ZnO was 400 nm in Fig. 2c and Fig. S3c, confirming the fitting results using Tauc plots. The Fermi level (*E*_f_) and valence band maximum (VBM) could be fitted from ultraviolet photoelectron spectra (UPS). Combining the results of ultraviolet photoelectron spectroscopy and absorption spectroscopy measurements, the conduction band minimum (CBM), *E*_f_, and VBM of F@ZnO were determined as − 4.16, − 4.40, and − 7.52 eV, respectively. Employing the values of CBM, *E*_f_, VBM of PbSe CQD active layer and EDT-PbS CQDs from the literature [3, 4], energy alignment of the device is shown in Fig. 5b. Compared with the band structure of SG ZnO [38], F^−^passiveted ZnO had deeper conduction and valence bands. F@ZnO with optimized band alignment could easily extract photogenerated electrons from the CQD active layer. Additionally, the upper shift of *E*_f_ in F@ZnO increased the open-circuit voltage (*V*_OC_) and depletion region width of the device. Benefiting from these effective optimizations, the device using F@ZnO as ETLs exhibited a superior power conversion efficiency (PCE) of 10.04%, compared to 9.18% for the device with SG ZnO. The EQE spectra demonstrated that the device with F@ZnO ETLs had stronger electron extraction ability than that of device with SG ZnO ETLs. The main reason was that the device with F@ZnO ETLs had wider depletion width.

## Conclusion

In summary, PbSe CQD solar cells with an improved performance were developed by employing F-passivated ZnO as ETLs. Owing to the strong binding ability of F to Zn and small radius of F anions, the oxygen vacancies and interstitial zinc in ZnO could be largely eliminated. Our control experiments showed that the passivation effect of F-passivated ZnO prepared from CBD method could not be simply repeated by using NH_4_F and NaF for surface treatment, indicating that the reported protocol was an efficient approach for the surface passivation of ZnO. Additionally, compared with the band structure of SG ZnO, F@ZnO possessed a favorable band alignment with deeper CB level and an upper shift of the *E*_f_, resulting in an increase of electron extraction efficiency from the CQD active layer. With these improvements, PbSe CQD solar cells using F@ZnO as ETLs showed a significant increase PCE of 10.04%, comparatively 9.4% higher than that of devices using SG ZnO as ETLs (9.18%). We are optimistic that this CBD-based interfacial engineering method has the potential for CQD applications, including photovoltaics, photodetectors, light emitting diodes, and flexible devices.

## Experimental section

### Materials

Lead oxide (PbO, 99.9%) and 1-octadecene (ODE, > 90%), cadmium oxide (99.99%, metals basis), selenium powder (Se, ≥ 99.99%, ≥ 200 mesh), N,N-dimethylformamide (99.8%, anhydrous), butylamine (BTA, ≥ 99%) were bought from Aladdin. Oleic acid (OA, 90%) and Bis(trimethylsilyl) sulfide (TMS, 98%) were bought from Sigma-Aldrich. Lead iodide (99% +) and lead bromide (99%) were purchased from Adamas. Zinc nitrate (Zn(NO_3_)_2_, 99%), zinc acetate dehydrate (Zn(Ac)_2_·2H_2_O, 99%), ammonium citrate (> 99%), NH4F (> 99%), ethanolamine (> 99%), 2-methoxyethanol (> 99%), octane (≥ 95%), acetone (≥ 99.5%) and methanol (≥ 99.7%) were bought from Sinopharm reagents. All solvents and materials were used directly without further purification.

### Preparation of sol-gel ZnO

1.5 g Zn(Ac)_2_·2H_2_O, 400 μL ethanolamine, and 20 mL 2-methoxyethanol were loaded in a 25 mL vial. The mixture was vigorously stirred at 60 for 10 h until hydrolysis was completely reacted in air condition.

### Preparation of ZnO seed layer

After SG ZnO precursor solution was prepared, several drops of SG ZnO were dropped onto the clean ITO substrate and spin coating at 4000 r/min for 30 s. Then, the ZnO seed layer was placed onto a hot plate initially at 100 ℃. 7 min later, the temperature of hot plate increased to 320 ℃. After the seed layer was annealed at 320 ℃ for 13 min, the hot plate was turned off and naturally cooled down to room temperature.

### Preparation of F-passivated ZnO

First, 1.734 g Zn(NO_3_)_2_ was dissolved in 91 mL DI water, following by addition of 9 mL ammonia into the Zn precursor solution. Then, 0.109 g ammonium citrate was dissolved in 15 mL DI water. 0.236 g NH_4_F was dissolved into 85 mL DI water. Subsequently, the above solutions were mixed together in a beaker and moved into a CBD environment at 70 ℃. The ZnO seed layer/ITO was immersed into the mixed solution for the growth of desired thickness of F@ZnO. Finally, the sample was taken out, washed by DI water, and dried for the fabrication of solar cells.

### Fabrication of PbSe CQD solar cells

The PbSe active layers were prepared by directly depositing PbSe CQD inks onto F@ZnO/ITO in glove box. The synthesis process of PbI_2_-capped PbSe CQD inks were described in the previous reports [4]. Then, EDT-treated PbS CQDs were coated on PbSe CQD layers. Finally, ~ 80 nm Au electrodes were deposited through thermal evaporation for solar cell fabrication.

### Material characterizations

XPS and UPS measurements were carried out with the equipment of Thermo Fisher Escalab 250Xi to investigate the chemical components and band alignment of F@ZnO. The phase structure of F@ZnO was measured by XRD equipment with a Bruker D8 Advance diffractometer with Cu Kα radiation,* λ* = 1.54 Å. Photoluminescence (PL) spectroscopy was measured using LabRAM HR800. UV–Vis absorption spectra were measured at room temperature using a PerkinElmer spectrophotometer (Lambda 950 using integrating sphere). The surface morphology of F@ZnO was characterized using an Atomic Force Microscope (SPM9700). The cross section of PbSe CQD solar cells was measured using scanning electron microscope (FE-SEM, FEI NOVA NanoSEM 450).

### Device characterizations

The conductivity of F@ZnO was calculated by extracting data from current–voltage curves, measured by Agilent B1500A. Keithley 2400 digital source meter was used to measure the PCE of PbSe CQD solar cells in air at room temperature. The illumination of simulated AM 1.5G (100 mW/cm^2^) was generated by xenon light source (Oriel, Model 9119, Newport). In order to make the results accurate, the device was covered by a metal mask with an effective area of 0.0414 cm^2^ during PCE measurement. For EQE test, Keithley 2400 source meter and light source, 300 W xenon lamp (Oriel, 69 911, Newport), were carried out. The light intensities and wavelengths were calibrated by a reference standard silicon photodetector (Newport 818-UV).

### Supplementary Information

Below is the link to the electronic supplementary material.

Supplementary file1 (PDF 1289 KB)

## Data Availability

The data that support the findings of this study are available from the corresponding authors, upon reasonable request.
