# Supplementary material for: Fluoride passivation of ZnO electron transport layers for efficient PbSe colloidal quantum dot photovoltaics
Source: Front Optoelectron. 2023 Oct 27;16(1):28. doi: 10.1007/s12200-023-00082-3 (PMC10611680; doi:10.1007/s12200-023-00082-3)
Supplement: Supplementary file 1 — Supplementary file1 (PDF 1289 KB) [file 12200_2023_82_MOESM1_ESM.pdf]

ASSOCIATED CONTENT

## Supporting Information

# Fluoride passivation of ZnO electron transport layers for efficient PbSe colloidal quantum dot photovoltaics

Jungang He,[*, †, §] You Ge,[†] Ya Wang,[§] Mohan Yuan,[†] Hang, Xia,[†] Xingchen Zhang,[†] Xiao Chen,[†] Xia Wang,[†] Xianchang Zhou,[†] Kanghua Li,[*, §] Chao Chen,[§] Jiang Tang[§]

† Hubei Key Laboratory of Plasma Chemistry and Advanced Materials, Hubei Engineering Technology Research Center of Optoelectronic and New Energy Materials, School of Materials Science and Engineering, Wuhan Institute of Technology, Wuhan 430205, Hubei, P. R. China

§ Wuhan National Laboratory for Optoelectronics (WNLO), School of Optical and Electronic Information, School of Integrated Circuits, Huazhong University of Science and Technology, Wuhan 430074, Hubei, P. R. China

KEYWORDS: Zinc oxide, surface passivation, band alignment, quantum-dot solar cells

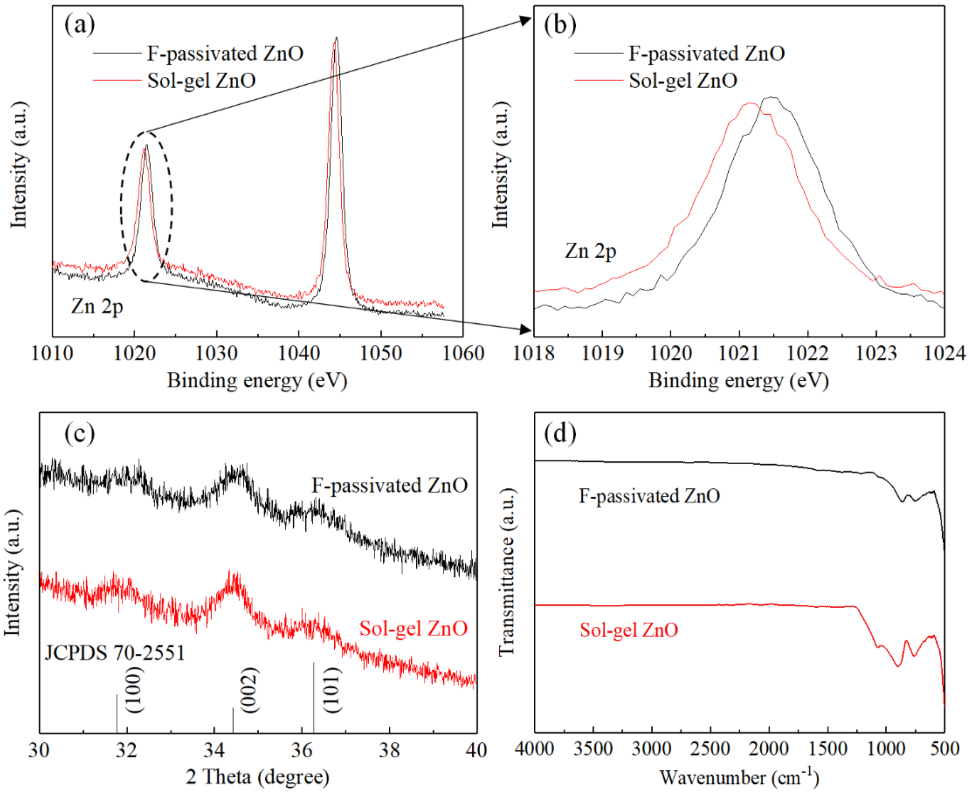


Fig. S1 (a) Zn 2p XPS of F-passivated ZnO and sol-gel ZnO. (b) Magnified Zn 2p XPS of F-passivated ZnO and sol-gel ZnO. (c) XRD patterns of ZnO with and without F passivation. (d) FTIR spectra of ZnO with and without F passivation.

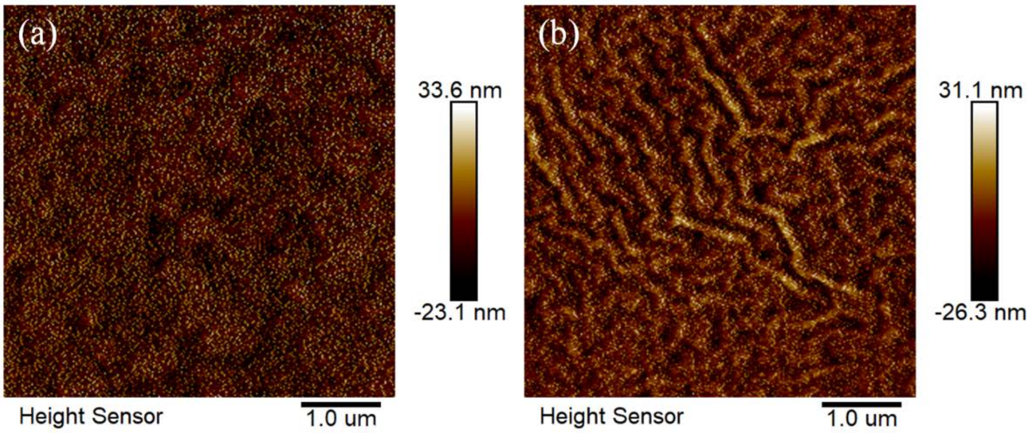


Fig. S2 Atomic force microscope characteristics. (a) F-passivated ZnO. (b)Sol-gel ZnO.

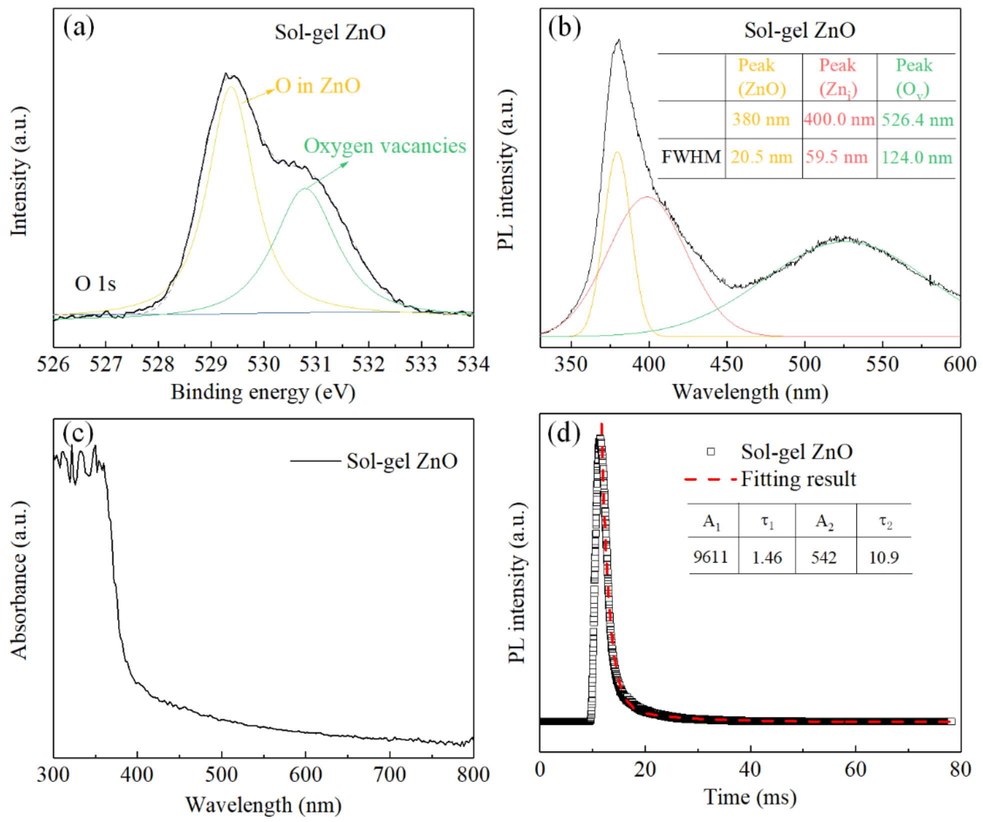


Fig. S3 (a) O 1s XPS of sol-gel ZnO. (b) PL spectra of sol-gel ZnO. The inserted table is the fitting FWHM results of ZnO defects. (c) Absorbance spectra of sol-gel ZnO. (d) Transient PL spectra of sol-gel ZnO.

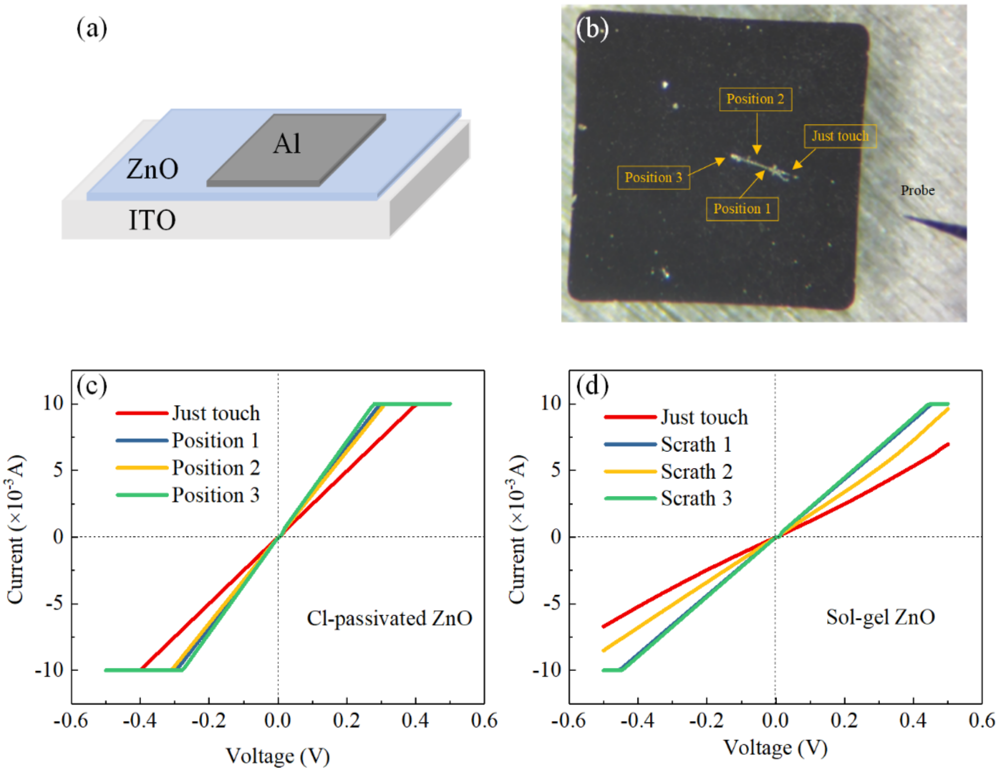


Fig. S4 (a) Schematic diagram of ZnO resistor for conductivity measurement. (b) Photograph of ZnO resistor measured at different position. (c) and (d) Current-voltage characteristics of Cl-passivated ZnO and sol-gel ZnO.

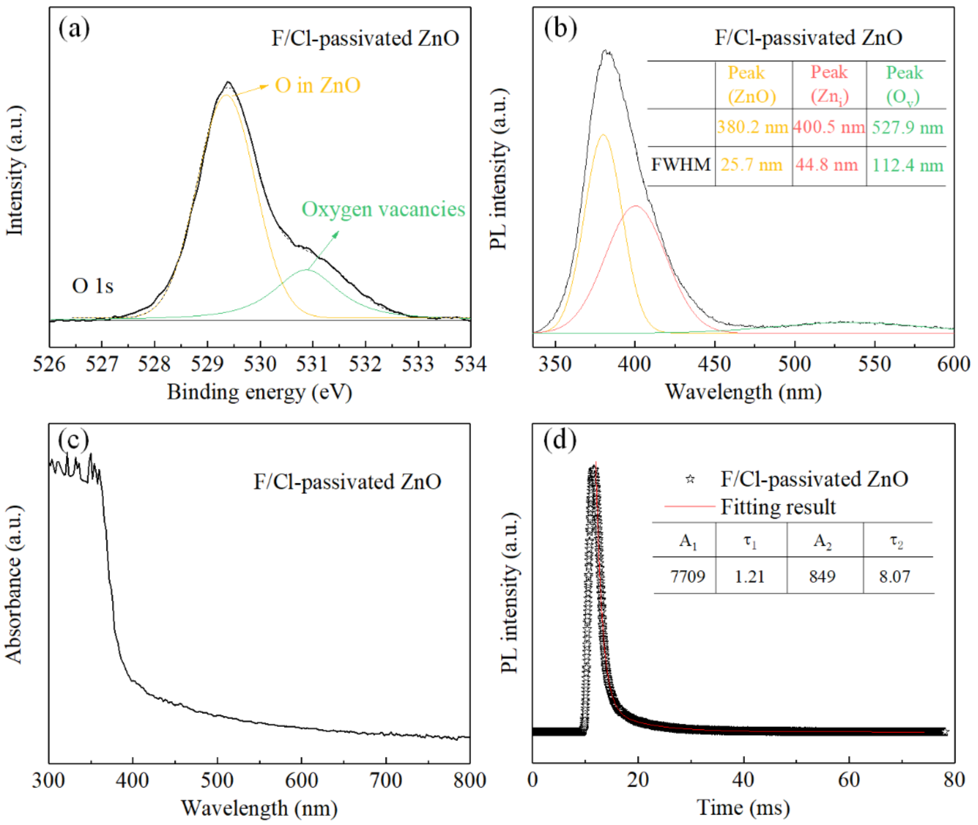


Fig. S5 (a) O 1s XPS of F/Cl-passivated ZnO. (b) PL spectra of F/Cl-passivated ZnO. The inserted table is the fitting FWHM results of F/Cl-passivated ZnO defects. (c) Absorbance spectra of F/Cl-passivated ZnO. (d) Transient PL spectra of F/Cl-passivated ZnO.

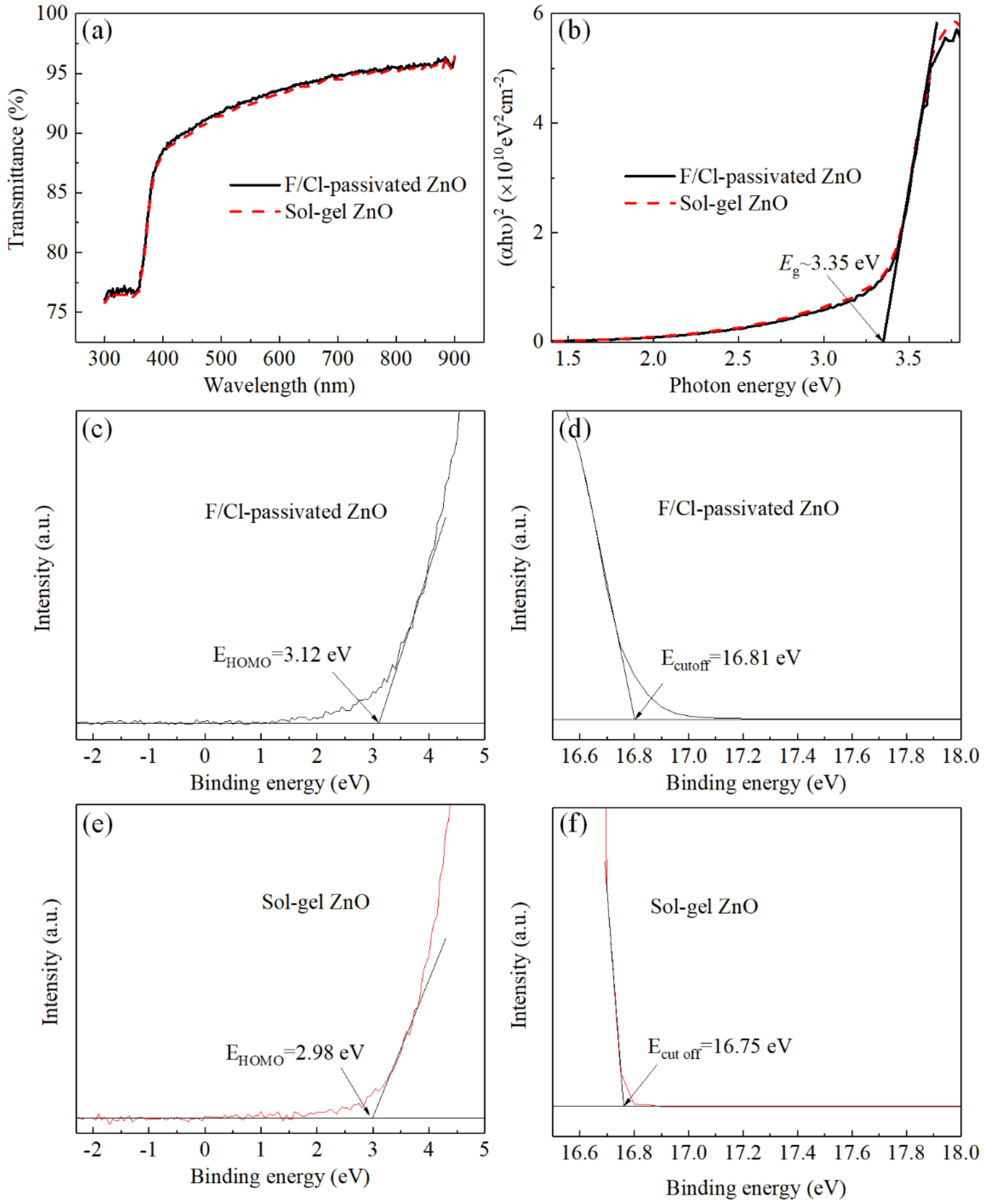


Fig. S6 Transmittance spectrum of F/Cl-passivated ZnO and sol-gel ZnO. (b) The Tauc plot of F/Cl-passivated ZnO and sol-gel ZnO. (c) and (d) Fermi level and secondary electron cutoff region of F/Cl-passivated ZnO. (e) and (f) Fermi level and secondary electron cutoff region of sol-gel ZnO.
